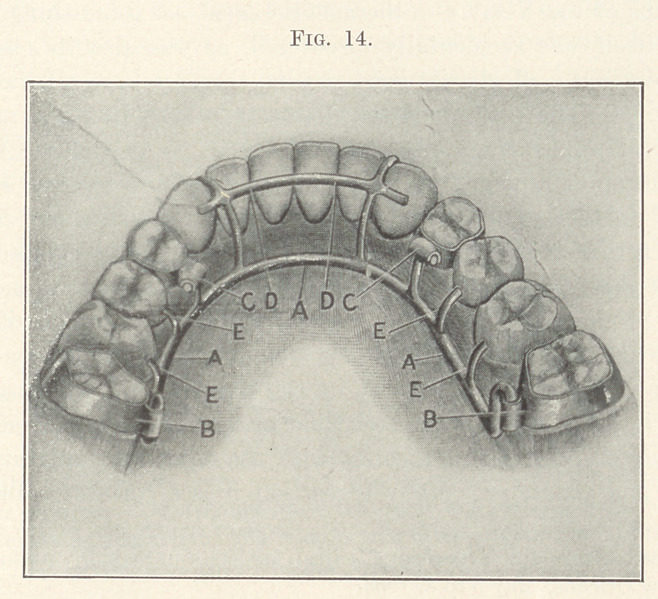# Treatment of Protruding and Receding Jaws by the Use of the Intermaxillary Elastics

**Published:** 1904-05

**Authors:** H. A. Baker

**Affiliations:** Boston, Mass.


### TREATMENT OF PROTRUDING AND RECEDING JAWS BY THE USE OF THE INTERMAXILLARY ELASTICS.[1]

BY DR. H. A. BAKER, BOSTON, MASS.

To my mind, of all the authors who have written upon the subject of Orthodontia, no one has given the term irregularities of the teeth so correct a definition as Edward H. Angle when he defined the term as malocclusion, for wherever you find irregular teeth you will find malocclusion. With perfect occlusion you will invariably find regular teeth.

Not only do I give him the credit of giving the most correct definition, but I also believe he has given to the profession the best appliance for its correction, although he disclaims the originality of it, his only claim being a modification of an old method.

I consider that this appliance can be adapted to the correction of a greater number of classes of malocclusion than any other one method. When the appliance is properly handled, better results can be obtained in the least possible time and with less discomfort to the patient.

The arches can be expanded or contracted, protrusion or recession of the jaws corrected, bringing into line in- and outstanding teeth, correcting lack of anterior occlusion by elongation of the teeth, and by the same means bringing into position partially erupted teeth; also rotation in all its forms and combinations can be accomplished to advantage with it, which practically covers all varieties of malocclusion.

A feature that I want to especially emphasize, and want all to appreciate, is that all of these deformities can be corrected at the same time, and, what is more, during the time that other appliances are correcting one. Take, for example, a case where the arch has to be expanded and there are crowded teeth, which generally occurs with contracted arches. The arch can be expanded in as short a time as any other device can accomplish the object; and while this is going on the twisted teeth can be rotated as readily as with a specific appliance for that purpose.

I hope I have made myself clear on this point, for, as I have above stated, it is an important feature.

---

[1] Read before The New York Institute of Stomatology, January 6, 1903.

Fig. 1.

Fig. 2.

FIG. 3.

FIG. 4.

Another advantage of this appliance and its modifications is the great amount of resistance that can be obtained, not only from the anchor teeth, but from all the teeth of the jaw which can be brought to bear on those that are to be moved, thus diminishing the danger of displacing or tipping the anchor teeth, which is apt to occur unless the operator is very skilful. I wish to go a step farther and say that the teeth of the opposite jaw from which the appliance is fixed will serve as resistance by the principle of inclined plane, a force that is of great importance in regulating, both to help or retard the work, according to whether the operator uses the force to work with him or ignores it, in which case it is very liable to work against him, and he wonders why he fails. Still another advantage is that if one or more of the teeth need more force applied, simply ligate them firmer or oftener than the others, or, if they are moving too rapidly, reverse the process and give them rest; thus the appliance is well under control.

One more feature is that after the deformity has been corrected, but the occlusion is not as good as desired (I refer especially to the bicuspid and molar region), by still keeping the appliance on enough freedom can be allowed the teeth for them to settle, so to speak, into position and adjust themselves. By keeping it on still longer it makes a very good temporary retainer. I have found that this is of great value in producing good occlusion.

We will endeavor to show how it is especially adapted to correct the class of cases that come under the title of this paper,—namely, protruding and receding jaws, which affect the expression of the face more than any other class. To be successful in correcting them, one should always have an ideal in mind and endeavor to approach it.

As we all very well know, the common device for correcting protrusion is the head-cap and the bit, which in the first place is very unsightly as well as uncomfortable; and secondly, my experience is that the patients object to it more than any other device connected with orthodontia. Because so many patients refuse to undergo the treatment with such an appliance, I have given the above question considerable study.

My youngest son was afflicted with a very pronounced case of protrusion and recession of the jaws, for which I studied out a course of treatment which I thought would be effective.

I brought my study model before the American Academy of

Dental Science and explained my method, after which I proceeded as I explained and carried the case through without a false step, if you will permit me to say so. I believe the illustrations will prove this to be true.

Case I.—When my youngest son was an infant of six weeks conditions were such that we were obliged to bring him up by artificial means, and he acquired the habit, so common among children, of keeping the rubber nipple in his mouth almost constantly. As a result, the gentle pressure of the soft rubber caused the deformity in his delicate jaws before being discovered, and after his permanent teeth had been erupted presented the appearance as shown in Fig. 1. Carefully considering the case, I decided to wait until just before the lower twelfth-year molars erupted, as shown in Figs. 2 and 3.

While studying these models, by sliding the lower jaw forward so that the sixth-year molars would be in a normal occlusion I found that with a very little spreading of the arches and slightly retracting the upper incisors I would get proper occlusion. By close observation we notice the deformity is confined to more of a recession of the lower jaw than protrusion of the upper. As the correction requires very little tooth movement and considerable forward bodily motion of the lower jaw, it was a great problem to me what force to apply to produce this result. I studied the case long and carefully, and it occurred to me that by using the Angle appliances in combination with elastic pressure applied in such a way as to obtain what we might call reciprocal anchorage, that is, to retract the superior incisors and at the same time bring the lower jaw forward to its normal position, we could obtain the desired results. To apply this theory I attached a moderately heavy elastic to each side of the lower appliance by slipping them over the ends of the tubes of the anchor bands, stretching them forward, and fastening to the superior expansion arch between the cuspids and laterals, as shown in Fig. 4. (Being a case that I could constantly watch I decided to make the trial.) I took my models before the American Academy of Dental Science and explained my method of procedure, requesting it to be put on record as a new device for correcting protruding and receding jaws. I commenced the case in the spring of 1893. I was astonished with the result. In two months' time the teeth were occluding in a normal position; but for fear that they might

Fig. 5.

Fig. 6.

Fig. 7.

Fig. 8.

FIG. 9.

FIG. 10.

Fig. 11.

Fig. 12.

return to their former position, I reduced the size and strength of the elastics and kept them in that way several months longer, and by so doing they settled into perfect occlusion.

The next step was to retain them. My experience with rubber retaining plates festooned around the teeth was so unsatisfactory that I thought out a method of retaining which I hoped would be more satisfactory. By considering carefully Fig. 5 we get a good idea of the superior retainer. The features of this device are, first, the extreme small amount of contact between the retainer and the enamel of the teeth, therefore improving the sanitary conditions which reduces the liability of causing decay to a minimum. The second feature of advantage is the amount of freedom that is allowed the teeth, permitting them to settle into proper occlusion, as well as the range of adjustment that is allowed by the alterations of the metallic spurs.

The retainer consists of a vulcanite suction-plate covering enough of the vault to insure its stability. From the plate radiate platinized gold spurs bearing at a single point against the cuspids, bicuspids, and molars. The incisors are held in their intended position by a wire of the same material passing around their labial surfaces and entering the palatal surface of the plate by being adjusted between the laterals and cuspids, care being taken not to interfere with the occlusion, either by the striking of the lower teeth or by separating the cuspid and lateral.

The lower retainer was made on the same general principle as the superior, excepting that suction was out of the question for holding the appliance in place. For this purpose I arranged a snap device similar to those used in coin-purses. We see in Fig. 6 that the first bicuspids are banded, and to these bands solder was flowed to thicken them, into which deep notches were filed for the purpose of holding spurs projecting from the plate. These spurs snap into the notches in the same manner that the snap fastener of a coin-purse works. The plate is further prevented from sinking into the soft tissue of the floor of the mouth by uprights from the heel of the plate hooking over into the crevices of the molars. To prevent backward movement of the plate spurs were extended from it on either side, resting on the mesial surface of the sixth-year molars. It is readily seen that both of these retainers can be removed by the patient for cleansing purposes, which I consider a great feature.

Fig. 7 shows occlusion after the retainers were adjusted. Fig. 8 shows the patient a few years later after the retainers were removed.

Case II.—Miss ——, aged between twenty-six and thirty, an extreme case of prognathism, as shown in Fig. 9. Fig. 10 shows model of case before beginning treatment, with the rear part of the lower model cut away to show the upper molars, which otherwise would have been concealed. This case is characterized by the extreme backward slant of the lower incisors so prevalent among cases of this class. There has been considerable controversy among the profession regarding the changes that take place during the treatment of a case of this description by means of the intermaxillary elastics, some holding that the change produced was due to tooth movement alone, while others were of the opinion that the results were obtained by the bodily retraction of the lower jaw itself. In order to settle the matter, in my own mind at least, I constructed a device at the suggestion of my son, Dr. Lawrence W. Baker, to record whatever changes took place. The construction and principle of the apparatus can be readily understood by studying Fig. 11, which shows the "recorder" in position. It consists of a metallic skeleton framework on a base of modelling compound covering the bony protuberances of the forehead and nose, to which indicators are attached to measure the relative movement of both teeth and jaw, the upper indicator measuring the tooth movement of the lower jaw and the lower one the movement of the jaw itself. It can be readily seen that this apparatus could be accurately placed in exact position at the various stages as the work progressed.

Fig. 12 shows the model of the completed case. It will be noticed that the six anterior teeth were carried forward to their normal position. It was proved by the indicator that the jaw was retracted by actual measurement one-quarter of an inch more than the teeth. This proved to me, beyond a question of doubt, that the lower jaw was retracted independently of the teeth.

Fig. 13 gives the change produced in the facial expression. The series of photographs illustrating the case shows clearly the importance of working for and getting the normal relations between the two jaws, and, furthermore, that the direction of the teeth and the proper modelling of the alveolar process all have their effect in producing harmony in the facial expression. The

FIG. 13.

FIG. 14.

method of retention employed in this case was based on the same principle that we have already considered in the first case,—that is, the retainers were constructed on the single contact point theory. However, they differed from the first, inasmuch as those were of a combination of vulcanite and metal, while these are entirely metallic, as shown in Fig. 14, giving the principle of construction much better than I can describe. I might, however, add that the anchor bands are of 22-carat gold, while the wirework is of platinized gold. The appliance is so made that it can be removed by springing the horizontal uprights out of the half-tubes attached to the bicuspid bands by the operator for adjustment and for cleansing purposes. I have treated many similar cases since adopting this method, but have shown to you the two extremes, which I trust has proved its efficiency.

## Figures and Tables

**Fig. 1. f1:**
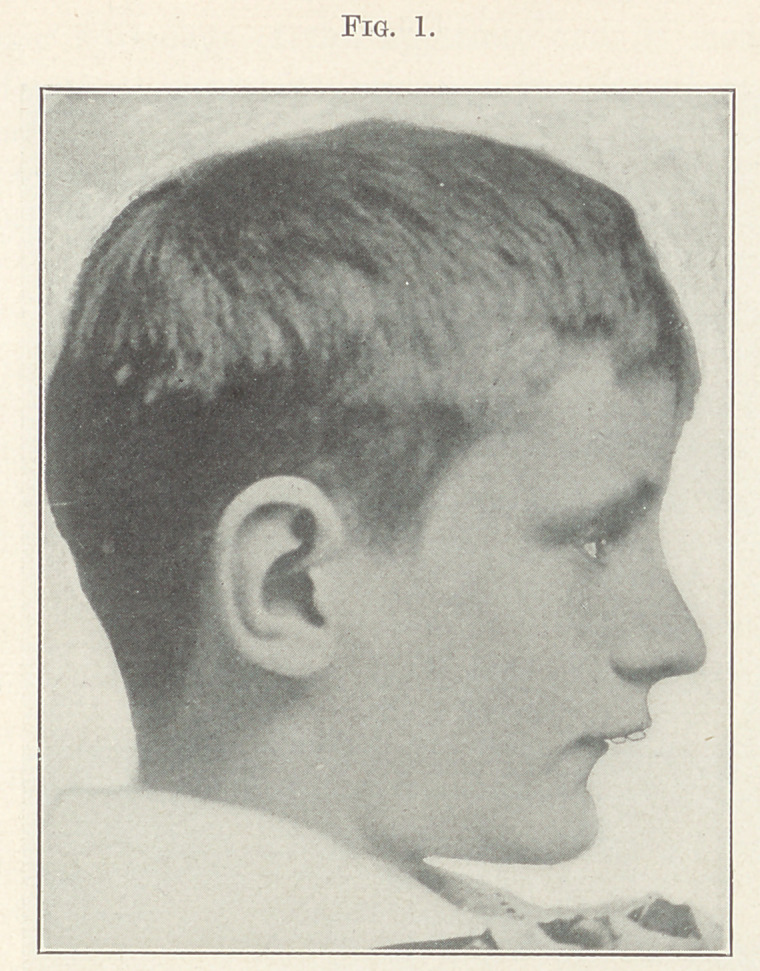


**Fig. 2. f2:**
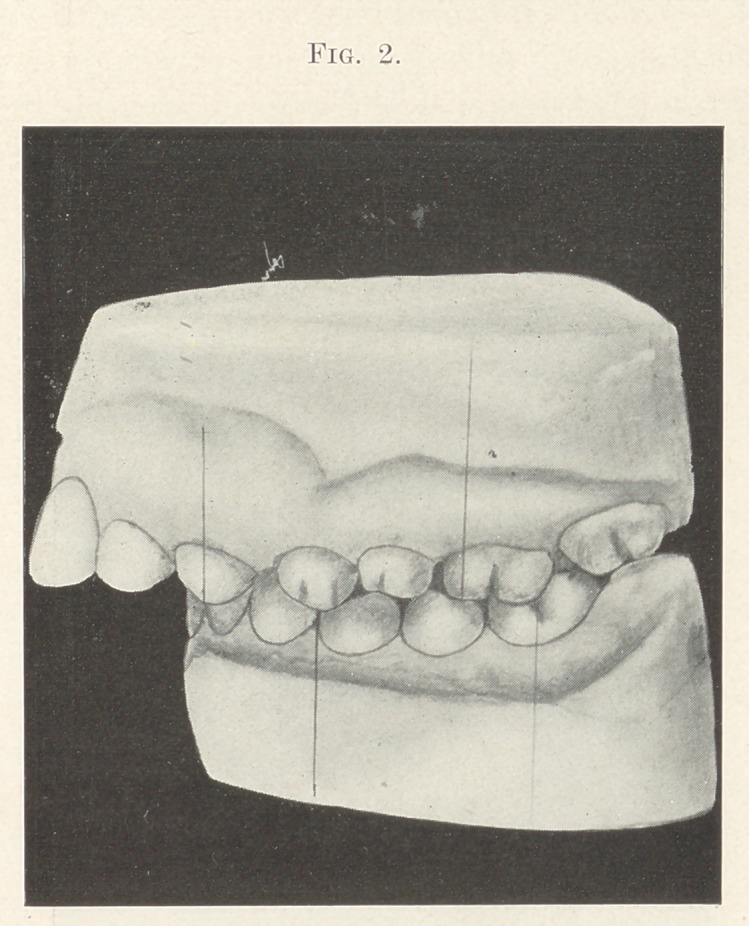


**Fig. 3. f3:**
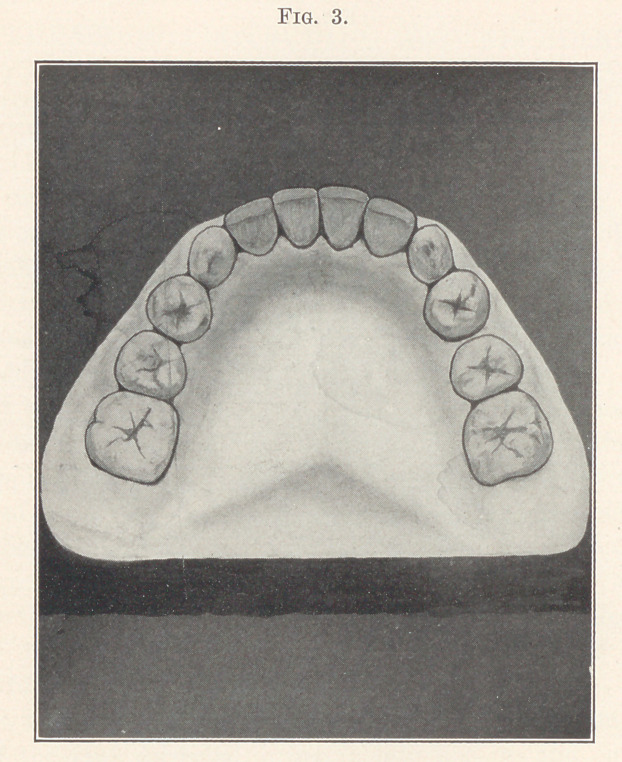


**Fig. 4. f4:**
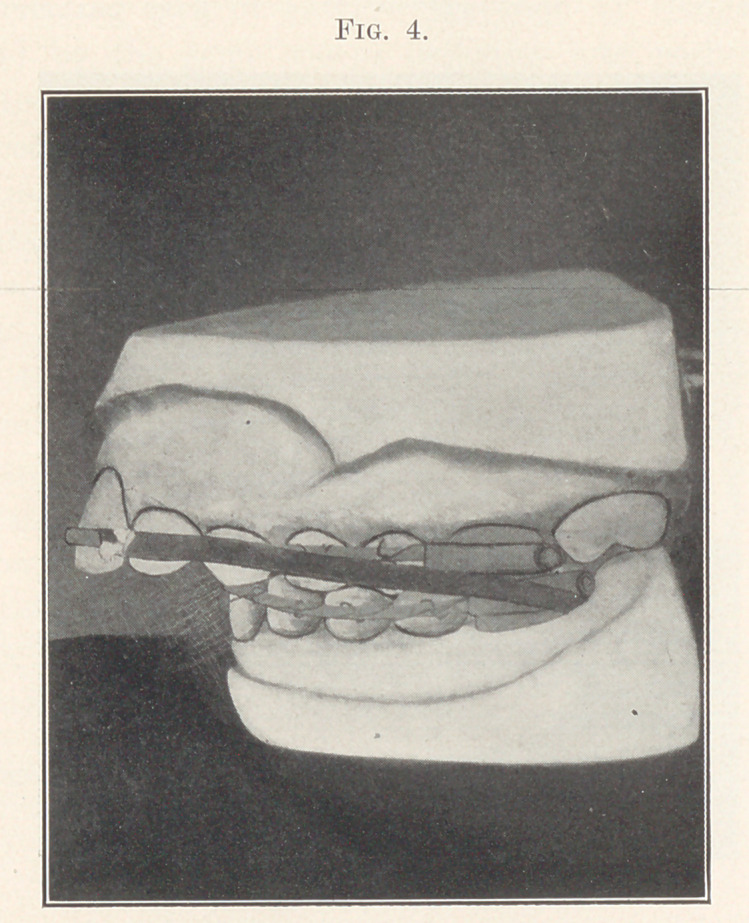


**Fig. 5. f5:**
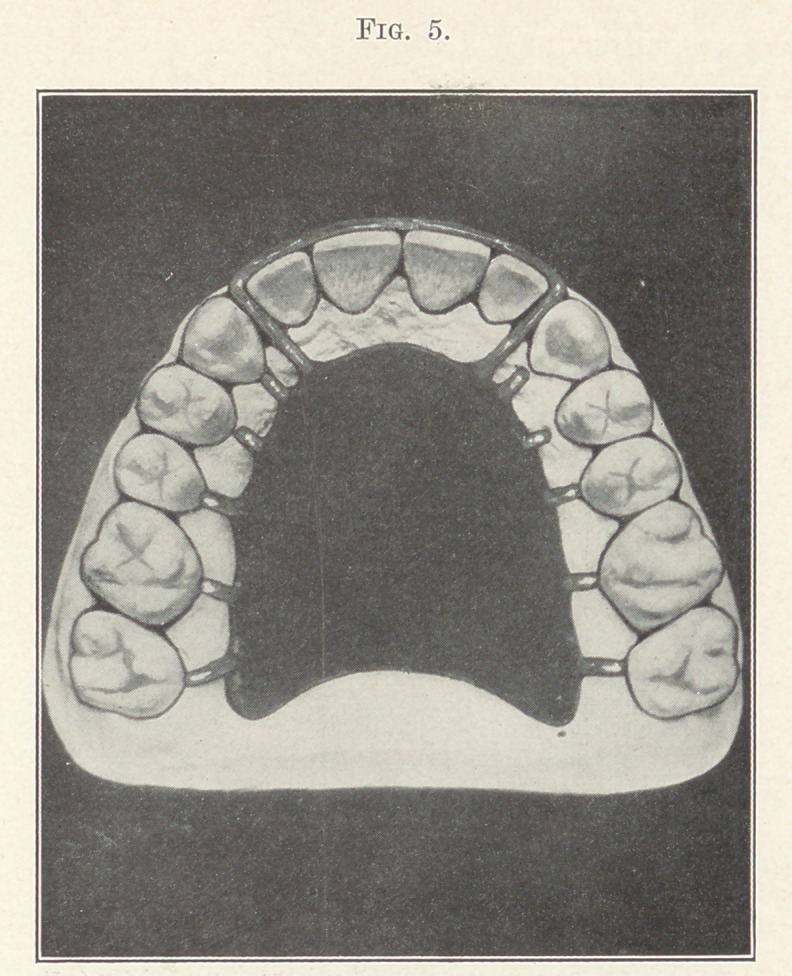


**Fig. 6. f6:**
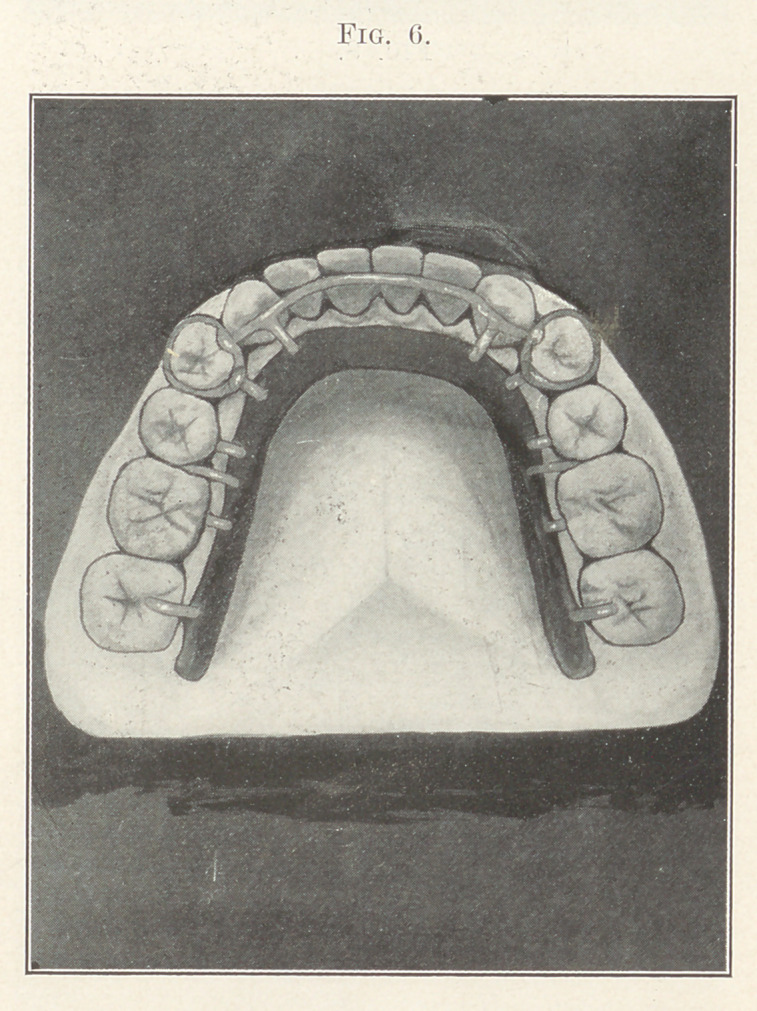


**Fig. 7. f7:**
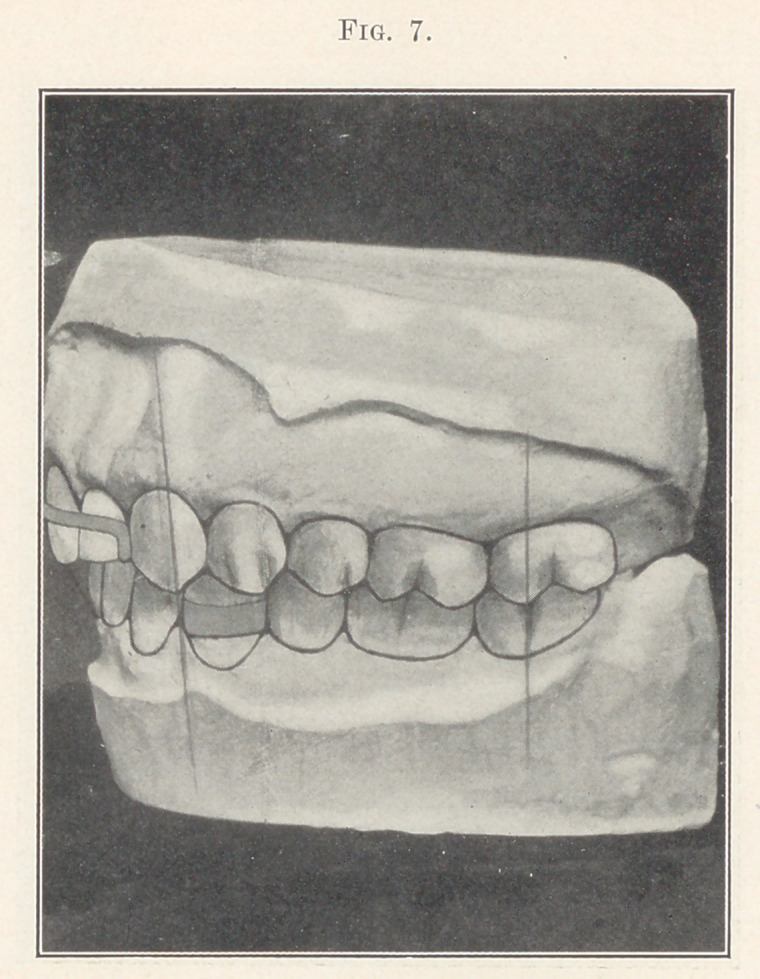


**Fig. 8. f8:**
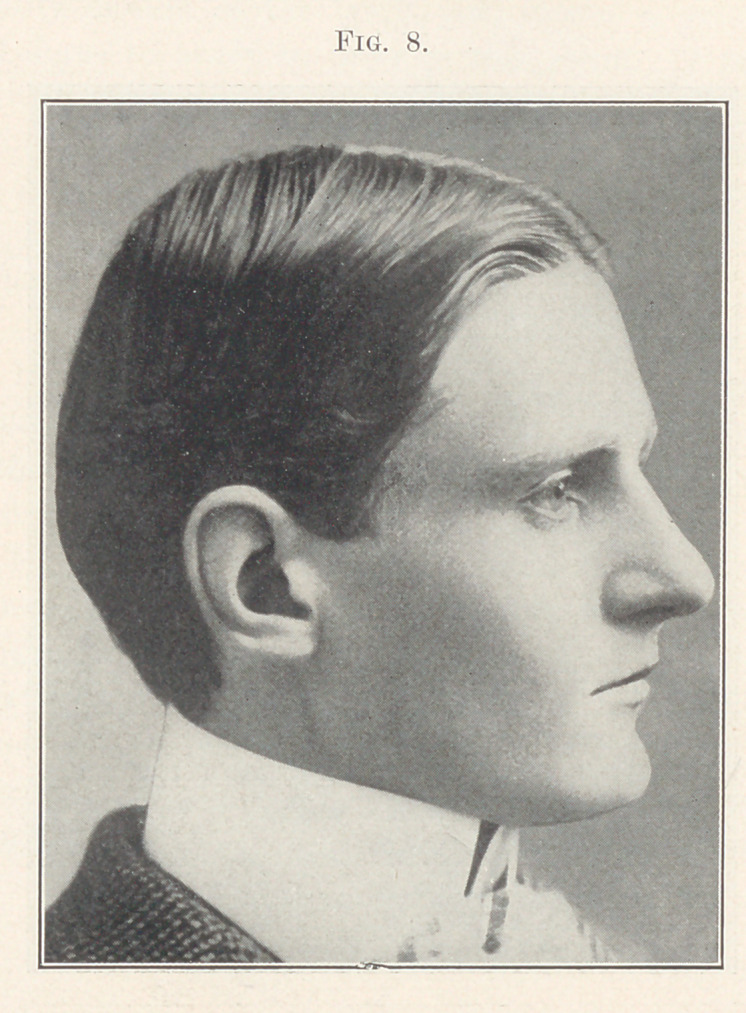


**Fig. 9. f9:**
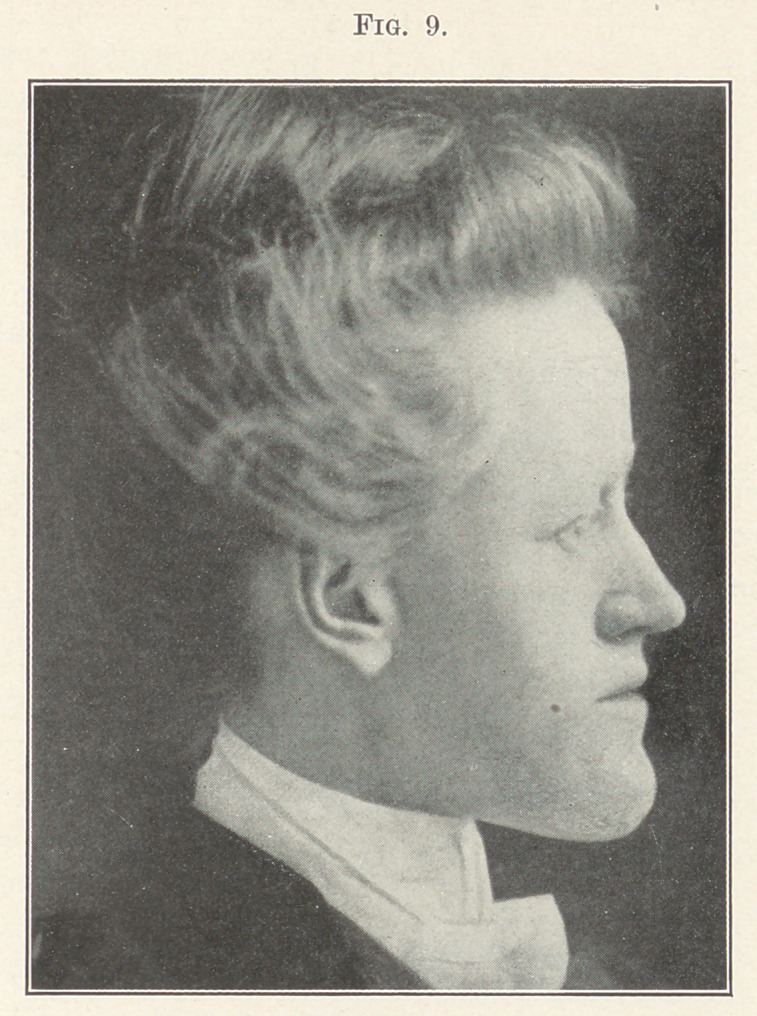


**Fig. 10. f10:**
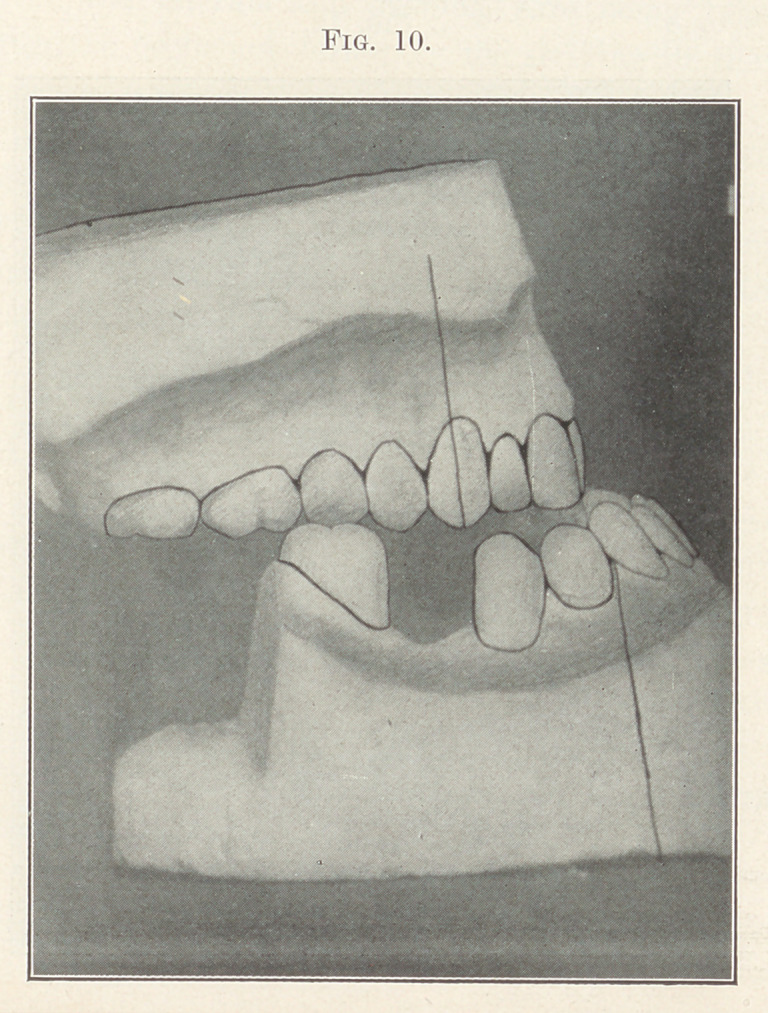


**Fig. 11. f11:**
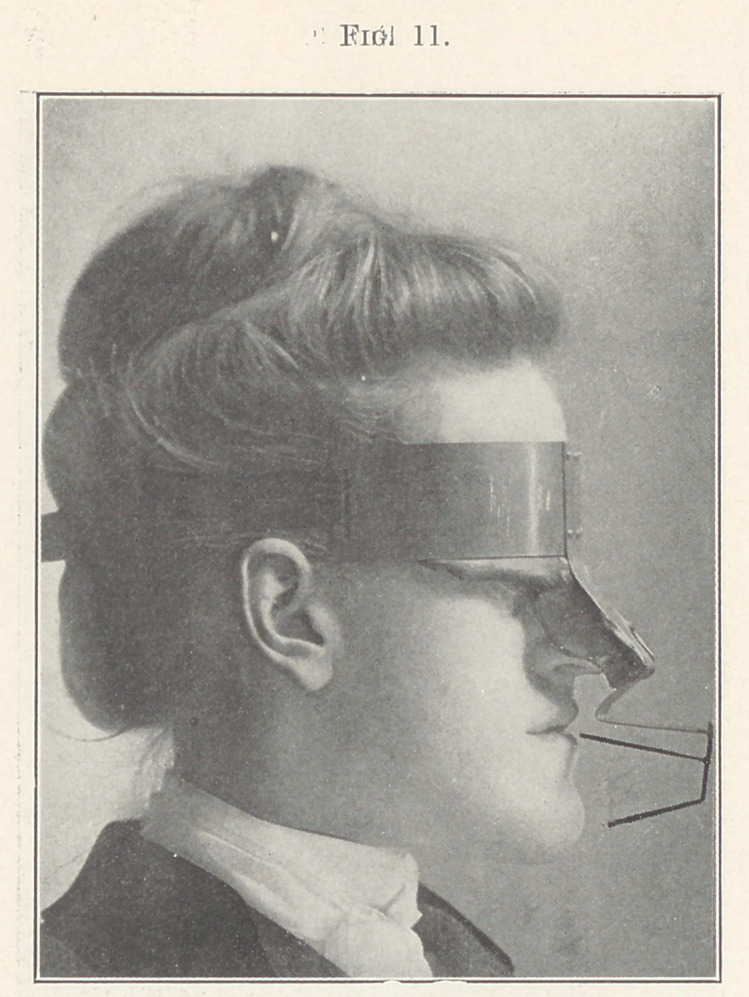


**Fig. 12. f12:**
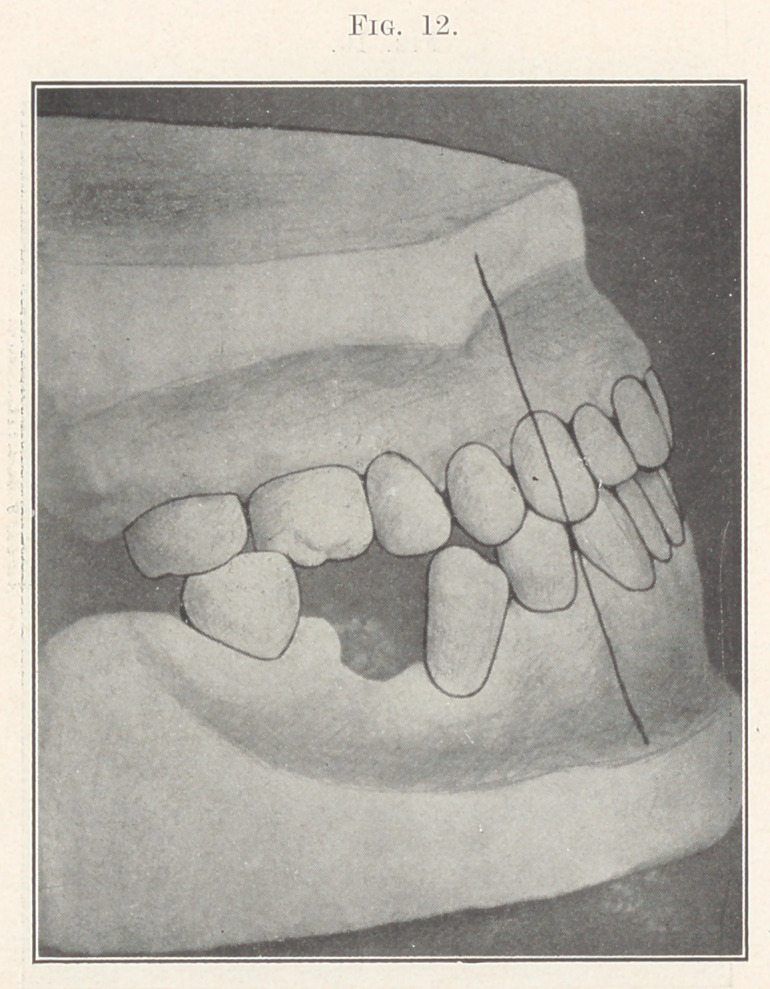


**Fig. 13. f13:**
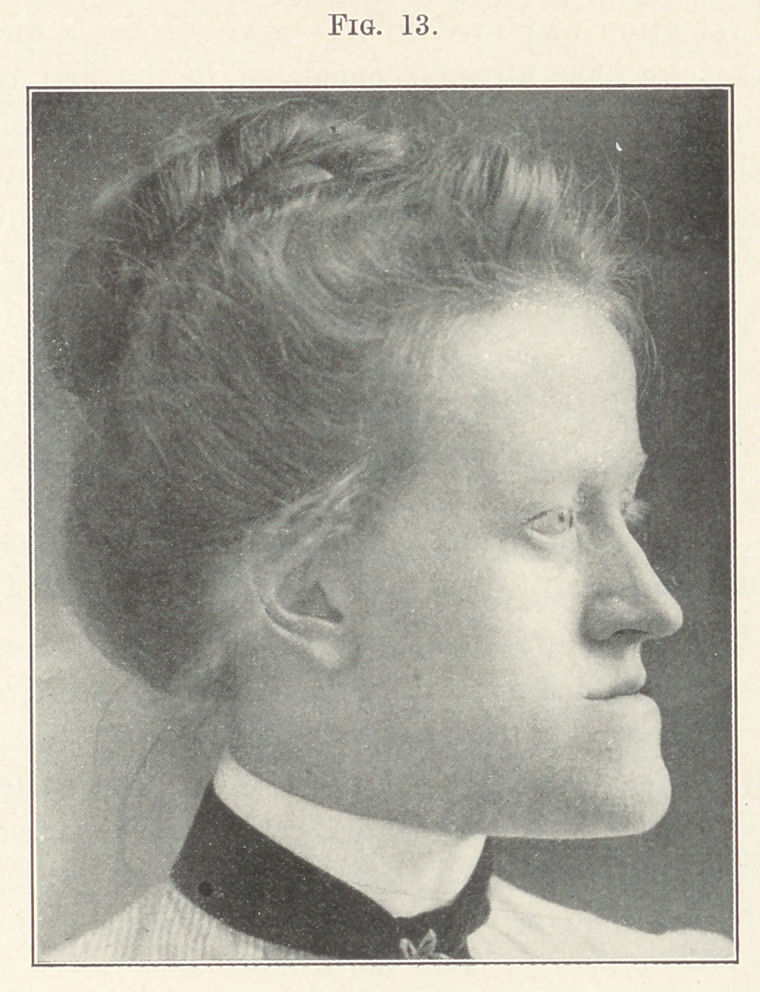


**Fig. 14. f14:**