# Correction Notice

**Published:** 1904-06

**Authors:** 


### CORRECTION NOTICE.

In Dr. H. A. Baker's paper on "Treatment of Protruding and Receding Jaws," published in the May number, there occurred a very regrettable transposition of two cuts,—Fig. 13 should have occupied the place filled by Fig. 9, and the latter in the former, showing the completion of the work and the correction of the irregularity.

No doubt all our readers made the correction for themselves, as the transposition was clearly made evident in the character of the illustrations.

